# The First Case of Native Mitral Valve Endocarditis due to *Micrococcus luteus* and Review of the Literature

**DOI:** 10.1155/2019/5907319

**Published:** 2019-12-04

**Authors:** Alisha Khan, Thu Thu Aung, Debanik Chaudhuri

**Affiliations:** State University of New York Upstate University Hospital, USA

## Abstract

Gram-positive cocci species, notably *Staphylococcus*, *Streptococcus*, and *Enterococcus* account for 80 to 90% of infective endocarditis cases. HACEK microorganisms (*Haemophilus* spp., *Aggregatibacter actinomycetemcomitans*, *Cardiobacterium hominis*, *Eikenella corrodens*, and *Kingella kingae*) account for approximately 3% of cases and *Candida* species account for 1-2% of cases. *Micrococcus luteus* is a rare cause of endocarditis. To our knowledge, only 17 cases of prosthetic valve endocarditis have been described due to *M. luteus* and a single case of native aortic valve endocarditis has been described. The following case is the only documented case of native mitral valve endocarditis. A review of the literature pertaining to Micrococcus endocarditis was performed to further characterize the entity.

## 1. Case Presentation

A 67-year-old gentleman presented to our hospital with complaints of dyspnea and orthopnea. His past medical history included diabetes, hypertension, three cerebrovascular accidents, peripheral vascular disease status post right below knee amputation (BKA), and moderate-to-severe mitral valve insufficiency. He was admitted with acute on chronic diastolic heart failure and was started on a bumetanide infusion. Of note, the patient's BKA was three months prior to admission and was complicated by bacteremia and sepsis.

On admission, his vital signs were stable and within normal limits. He was intermittently noted to be tachypneic with his respiratory rate reaching 26 breaths per minute. Physical examination was remarkable for jugular venous distention, grade 4/6 systolic murmur best heard at the mitral position, decreased lung breath sounds at the bases, and trace pedal edema. Complete blood count and comprehensive metabolic panel laboratory values were within normal limits. Pro b-type natriuretic peptide level was elevated at 5309 pg/mL. Two sets of blood cultures showed no growth at 5 days. Chest radiograph revealed large bilateral pleural effusions. Transthoracic echocardiogram revealed prolapse of the anterior mitral leaflet with moderate-to-severe mitral regurgitation, hyperdynamic left ventricular (LV) systolic function with an ejection fraction between 65 and 70%, no evidence of vegetation, and an estimated pulmonary artery systolic pressure of 71 mmHg with moderate-to-severe tricuspid regurgitation. Preoperative left heart catheterization showed 80-90% stenosis of the diagonal artery and elevated left ventricular diastolic pressure. Snapshot hemodynamic recordings of the aortic pressure revealed 109/70 mmHg (mean 86 mmHg), whereas the left ventricle measured 110/11 mmHg (mean 27 mmHg).

The patient was diuresed over several days with partial relief of his respiratory symptoms. Cardiac catheterization showed occlusion of one of the diagonal branches that was not amenable to endovascular intervention. The cardiothoracic surgery team evaluated the patient for replacement of his mitral valve. He was brought to the operating room where he underwent cardiopulmonary bypass and had his native mitral valve replaced with a 29-Medtronic Mosaic porcine bioprosthetic valve. Upon visual inspection, the native mitral valve was found to have fibrinopurulent exudate on the anterior and posterior leaflets. Surgical tissue cultures of the excised mitral valve grew *Micrococcus luteus*. Surgical pathology of the excised valve showed acute endocarditis with focal necrosis. Antibiotic susceptibility testing revealed that the *Micrococcus luteus* was sensitive to vancomycin, clindamycin, erythromycin, and penicillin. Replacement of his mitral valve and concurrent diuresis resulted in significant symptomatic and echocardiographic findings. Transthoracic echocardiogram performed approximately one week following valve replacement showed resolution of the mitral and tricuspid regurgitation as well as normalization of the pulmonary artery systolic pressure, indicating that the initial derangements noted on the echocardiogram were likely dynamic changes resulting from the mitral regurgitation. Ejection fraction at this time was noted to be 60-65%.

The patient was initially treated with empiric antibiotics including vancomycin, gentamicin, and rifampin. However, the patient developed acute kidney injury due to acute tubular necrosis from vancomycin and gentamicin and was subsequently transitioned to rifampin 300 mg by mouth every 12 hours and daptomycin 8 mg/kg intravenously every 48 hours at 100 mL/hour over 30 minutes to complete his treatment course for a total of six weeks of antibiotics. The patient was subsequently discharged to cardiac rehabilitation while receiving a total of six weeks of intravenous antibiotics to treat for endocarditis from the date of his mitral valve replacement.

Since treatment for his infective endocarditis, the patient's bioprosthetic valve appeared to have been functioning well for several months. However, approximately nine months after replacement, he was diagnosed with end-stage renal disease. Due to the associated fluid overload, his most recent echocardiogram shows that he has recurrent moderate mitral valve regurgitation; however, the prosthetic valve is well seated and does not rock. He is currently undergoing preparation for hemodialysis.

## 2. Discussion

*Micrococcus* species are Gram-positive, catalase-positive, oxidase-positive, nonmotile, and nonspore-forming cocci that comprise oropharyngeal and skin flora and rarely cause disease [1]. In fact, *M. luteus* used to be considered a nonpathogenic saprophyte or pure contaminant.

*Micrococcus* species, along with coagulase negative-staphylococci, viridans group streptococci, *Propionibacterium acnes*, *Corynebacterium* spp., and *Bacillus* spp., are the microorganisms the College of American Pathologists considers to be the most common blood culture contaminants when isolated from one out of two or three blood sets [2, 3]. However, in rare circumstances, *Micrococcus* spp. may be the causative organism for infectious diseases such as endocarditis. The first documented disease due to the organism was septic shock in 1978, when it was simultaneously recovered from a patient's blood cultures and gallbladder isolate [4]. In our case, the patient's diagnosis of infective endocarditis was confirmed by histology of the surgical specimen and the presence of new valvular regurgitation, according to the Modified Duke Infective Endocarditis Criteria.

The five species in the *Micrococcus* genus include *M. luteus*, *M. lylae*, *M. antarcticus*, *M. endophyticus*, and *M. flavus*. *Micrococcus luteus* is an obligate aerobe and has one of the smallest genomes of free-living actinobacteria sequenced to date, comprised of a single circular chromosome of 2,501,097 base pairs encoding 2,403 proteins [5].

*M. luteus* is a rare cause of endocarditis. Although low in virulence and usually sensitive to penicillin, it may colonize the surface of heart valves in immunosuppressed patients [1]. It has now been proposed as the pathogenic organism responsible for bacteremia, ventriculitis, peritonitis, pneumonia, endophthalmitis, keratolysis, and septic arthritis in isolated case reports [6].

In November 2018, a case of bacteremia due to *Micrococcus luteus* was described in an immunocompromised patient with a central venous catheter [7]. In December 2018, *M. luteus* was found to be the cause of a brain abscess in an immunocompromised patient with systemic lupus erythematosus who was being treated with immunosuppressive therapy for lupus nephritis [8]. Our patient's uncontrolled diabetes mellitus, with an HbA1c level of 7.4% on admission appears to have been his only known predisposing factor for immunosuppression, which would make him more susceptible to infection with a low virulence organism such as *M. luteus*.

In a review of the literature published by Miltiadous and Elisaf in 2011, only 17 cases of native valve endocarditis secondary to *M. luteus* had been reported and all involved prosthetic valves. A single case of native aortic valve endocarditis due to *M. luteus* has been described in an immunosuppressed patient [9]. To our knowledge, there are no case reports describing isolated native mitral valve endocarditis due to *M. luteus*.

We suspect that our patient became bacteremic with *M. luteus* during his BKA surgery three months prior to admission. Interestingly, in the case of native aortic valve endocarditis described by Miltiadous and Elisaf, the authors presumed that orthopedic surgery (total knee replacement) three weeks prior to admission was the source of bacteremia [9]. This supports the theory that in order for low virulence organisms such as *M. luteus* to become pathogenic and cause serious and invasive infections such as endocarditis, a significant bacterial load is required.

Due to the rarity of this microorganism as a cause for infective endocarditis, the optimal therapeutic regimen remains undefined. Of note, in the case of native aortic valve endocarditis secondary to *M. luteus* mentioned above, a treatment regimen consisting of vancomycin, gentamicin, and rifampicin for four weeks was *not* successful and the patient ultimately required aortic valve replacement [9]. Our patient who was treated with rifampin and daptomycin in addition to mitral valve replacement appears to have been successfully treated thus far.
